# Health Equity and Its Economic Determinants (HEED): protocol for a pan-European microsimulation model for health impacts of income and social security policies

**DOI:** 10.1136/bmjopen-2022-062405

**Published:** 2022-07-19

**Authors:** Srinivasa Vittal Katikireddi, Daniel Kopasker, Anna Pearce, Alastair H Leyland, Mikael Rostila, Matteo Richiardi

**Affiliations:** 1 MRC/CSO Social and Public Health Sciences Unit, University of Glasgow, Glasgow, UK; 2 Department of Public Health Sciences, Stockholm University, Stockholm, Sweden; 3 Centre for Microsimulation and Policy Analysis, University of Essex, Colchester, UK

**Keywords:** epidemiology, public health, mental health

## Abstract

**Introduction:**

Government policies on taxation and social security are important determinants of population health outcomes and health inequalities. However, there is a shortage of evidence to inform policymakers of the health consequences of such policies. The Health Equity and Its Economic Determinants project aims to assess the potential impacts of different taxation and social security policies across Europe on population health and health inequalities using a computer-based simulation that provides projections over multiple health domains.

**Methods and analysis:**

In the first phase, key input parameters for the model will be estimated using estimation techniques that control for the effects of prior exposure on time-varying confounders and mediators (g-methods). The second phase will involve developing and validating the microsimulation model for the UK. Policy proposals, developed with policymakers, will be simulated in the third phase to investigate the impacts of income tax and social security changes on population health and health inequalities. In the final phase, the microsimulation model will be extended across other European countries.

**Ethics and dissemination:**

This project will use deidentified secondary data for which ethical approval and consents were received by the original data collectors. No further ethical approval will be required for our main analytical datasets. Dissemination plans include academic publications, conference presentations, accessible policy briefings, mass media engagement and a project website. Both the syntax and the underlying synthetic data for the HEED microsimulation model will be made freely available through GitHub and the project website.

**Strengths and limitations of this study**

- Health Equity and Its Economic Determinants will be the first multicountry microsimulation policy model focusing on health and its economic determinants.
- The microsimulation model will be populated with health effects estimated using advanced epidemiological methods that control for prior exposure and time-varying confounding.
- The distributional effects of taxation and social security policies will be estimated using an established and widely used open-source tax-benefit microsimulation model (EUROMOD for EU countries, and UKMOD for the UK).
- Hypothetical policy scenarios, to be simulated within the model, will be developed with policymakers to ensure they answer relevant questions.
- The absence, in some European countries, of longitudinal data covering a sufficient period that are representative of the population and can be used to populate the microsimulation model will limit validation procedures for these countries.

## Introduction

Population health is strongly influenced by the social determinants of health.^1^ Government policies on income and social security are thought to be particularly important, with evidence suggesting investment in these areas provides greater health dividends than healthcare.^2^ Furthermore, such policies are of substantial importance for health inequalities.^3^ A ‘health in all policies’ (HiAP) approach encourages governments and others to actively consider the health consequences of broader policies such as these.^4 5^ However, a lack of directly actionable evidence hinders achieving a HiAP approach.^6^

A large body of evidence demonstrates the importance of economic determinants for overall population health and health equity.^7–9^ Economic development is consistently associated with improved life expectancy.^10^ Similarly, poverty is a well-established and potentially modifiable cause of poor health.^11^ However, quantifying these links is not straightforward. For example, there has been considerable interest in the health effects of economic recessions, with some aspects of health improving rather than worsening.^12–16^ Importantly, economic policy responses to recessions appear to modify health impacts substantially, suggesting that government economic policies have the potential to bring about large-scale changes in population health.^17–19^ More recently, there are concerns that stalling life expectancy in some countries might arise as a consequence of economic policies.^20–22^ A focus on the economic determinants of health requires a consideration of employment, as well as income. Being in paid work, rather than in unemployment, is associated with better health and might help reduce health inequalities.^23–26^ Policies to provide greater benefit income could therefore result in unintended adverse consequences if they alter labour market incentives, for example, if people stop working and experience some of the adverse health effects related to unemployment.

Tools to foster an integrated perspective to decision-making across health and economic policy have the potential to encourage a HiAP approach. A simulation model can provide a simplification of the real world that is useful for research, policy or practice. Simulation studies integrate existing evidence, real-world data and theory by taking these inputs and applying mathematical processes to create predictions. Microsimulation models do this using individuals and households as the unit of the analysis, hence allowing inequalities according to multiple individual-level characteristics to be studied.

In economics, tax-benefit microsimulation modelling has long been used to interrogate the complexity of income and welfare policy systems in high-income countries and to study their implications for economic inequalities.^27^ Their use is well established for informing public policy, with EUROMOD (a EU-wide static tax-benefit microsimulation model) being used by the European Commission to inform its decision-making. Despite the substantial consequences of income and social security policies for population health and health inequalities, models which combine disciplinary considerations from economics and public health are lacking.

Providing detailed evidence about policy implications requires an appreciation of the complexity that exists in relation to the economic determinants of health, with feedback loops, phase shifts and non-linearity potentially leading to unpredictable health outcomes.^28 29^ Changes in one policy can lead to a dynamic interaction with other public policies; for example, changes in the income tax threshold can influence entitlement for income supplementation schemes, which in turn affects take-home income in counterintuitive ways. Furthermore, policy effects differ across population subgroups; hence, impacts are heterogenous, potentially benefiting some groups while penalising others.^30^ Ultimately, predicting the overall health impacts of changes in the economic determinants requires the creation of a policy model to link policy changes to health impacts.

### Aim and objectives

The Health Equity and Its Economic Determinants (HEED) project aims to assess the potential impacts of different European taxation and social security policies on population health and health inequalities in the short, medium and long terms, using a dynamic microsimulation model. The specific research objectives are

1. To estimate the causal effects of changes in employment status and income on self-assessed physical health, mental health, life satisfaction and all-cause mortality.
2. To create projections for future health status, life satisfaction, mental health and all-cause mortality by age, sex and socioeconomic groups, for the UK over 1, 5 and 10 years.
3. To develop a dynamic, stochastic, discrete-time microsimulation model that predicts the health impacts of changes in taxation and social security policy for the UK and selected EU member states.
4. To estimate the impact of income tax and social security reforms in the UK on the aforementioned health outcomes over time horizons of 1, 5 and 10 years.
5. To estimate the impact of income tax and social security reforms on self-rated health, probable depression, life satisfaction and mortality across selected EU countries over 1 and 5 years.

## Methods and analysis

The HEED project will comprise four phases running sequentially. In the first phase, we will develop key inputs for the model. The second phase will involve developing the structure of the microsimulation model for the UK, including the creation of a representative synthetic population, projecting future outcomes and conducting a range of validation checks. The third phase will involve working with policymakers, predominantly in the UK, although input from selected policymakers in the EU will be sought, to develop realistic policy proposals to investigate the impacts of income tax and social security changes on population health and health inequalities. The final phase involves further development of the microsimulation model to study impacts across other European countries, making it the first multicountry policy model on health and its economic determinants. The HEED microsimulation model will be made freely available for reuse.

### Phase I: developing the model inputs

Our empirical analyses in phase I will draw on two existing datasets. The UK Household Longitudinal Study (UKHLS) will be used to derive effect estimates for self-reported health outcomes. UKHLS is a representative panel study of 40 000 UK households which collects annual information on individual and household characteristics from 2009 onwards. In addition to our analyses using UKHLS, we will conduct parallel analyses using Swedish register data to gain greater statistical power for some health outcomes. Health data include mortality records and prescriptions (such as psychotropic medications) data. Exposure variables will be available from the Longitudinal Database for Health Insurance and Labour Market Studies.^31^

Table 1 summarises the estimands of interest, outcomes and data sources for phase I. Key exposures are income and employment status. Outcome measures from the UKHLS include mental health (assessed by the 12-item General Health Questionnaire (GHQ-12)), health status (the Short Form 12-Item Survey (SF-12), which consists of two subscales: the Mental Component Scale and the Physical Component Scale) and self-rated health. Administrative data in the UK do not provide sufficient detail on employment and income and linkage to health records is not available. Therefore, Swedish register data will be used for analyses of all-cause mortality and mental health prescriptions, as well as allowing the consistency of effect sizes for other outcomes to be assessed, where possible.

**Table 1:** Potential sources of effect estimates for HEED simulation model parameter inputs

Estimands of interest	Outcomes	Datasets	Alternative sources
Employment status on mental health	GHQ-12, SF-12 (MCS)	UKHLS	Paul and Moser^58^ Thomas *et al*^59^ Flint *et al*^60^
Employment status on physical health	SF-12 (PCS) and SRH	UKHLS	McKee-Ryan *et al*^61^ Norström *et al*^62^
Employment status on mortality	All-cause mortality	Swedish register data, UKHLS	Roelfs *et al*^23^
Income on mental health	GHQ-12, SF-12 (MCS), prescriptions for psychotropic medications	UKHLS, Swedish register data	Systematic review by HEED research team^63^
Income on health status	SF-12 (PCS)	UKHLS	McCartney *et al*^64^
Income on all-cause mortality	All-cause mortality	Swedish register data, UKHLS	McCartney *et al*^64^

GHQ-12, 12-Item General Health Questionnaire; HEED, Health Equity and its Economic Determinants; MCS, Mental Component Score; PCS, Physical Component Score; SF-12, Short Form 12-Item Survey; SRH, self-rated health; UKHLS, UK Household Longitudinal Study.

We will use epidemiological techniques (g-methods) designed to address the time-varying nature of the exposures, mediators and outcomes, as well as compare our estimates against alternative approaches that are subject to differing assumptions (such as fixed effects regression). We will start by creating a directed acyclic graph for each outcome separately, but including both income and employment, specifying the direction of causality between variables at each discrete time step. Income, which might be affected by policy changes, will be considered as a mediator for some of the effects of employment status on health. To incorporate this mediation effect in the microsimulation model, we will estimate both the direct and indirect effects (via income) of employment status. Confounders will be categorised as measured (available in the data) or not, differentiating between potentially time-varying (eg, age, housing tenure, marital status and geographical region), including those which are affected by the exposure, and time-invariant (eg, sex, and ethnicity). Models will be stratified by sex, plus any further groupings deemed necessary to improve the microsimulation model (eg, age and education). Multiple imputation will be used to address item missingness and inverse probability weights to account for attrition. Where appropriate, new weights will be created to account for survival bias for outcomes other than mortality.

While g-methods are explicitly designed to provide causal effect estimates, a key assumption is no unmeasured confounding (including of baseline confounders).^32^ We will therefore triangulate our estimates, where possible, with those derived from approaches that do not rely on the measurement of baseline confounders but are subject to other assumptions (such as fixed effects regression).

An alternative approach to estimating causal effects is to study natural experiments where changes in the exposure have occurred for reasons other than experimental manipulation by researchers.^33 34^ We will compare our effect estimates with estimates from previous work where the health impacts of policy changes that affect income and/or employment status have been evaluated.^35–39^ We will also compare our estimates to those derived from available systematic reviews on the association between income and health. To improve the transparency of the modelling process, we will use the Grading of Recommendations Assesssment, Development and Evaluation (GRADE) Working Group methodology to assess and report the certainty of evidence for each effect estimate used in the model.^40 41^

### Phase II: developing the microsimulation model

In phase II, we will create a synthetic cohort representative of the UK population (eg, 3.5 million synthetic individuals nested within 1.9 million households for the year 2018, reflecting 10% of the population aged 25–64 years). We have focused on this age group because it captures the core working age population where potential labour market dynamics are important but excludes phases of the life course most related to education and retirement. However, we will consider expanding the age range of the population if that becomes feasible due to the creation of data resources by other researchers.^42^

**Figure 1:**
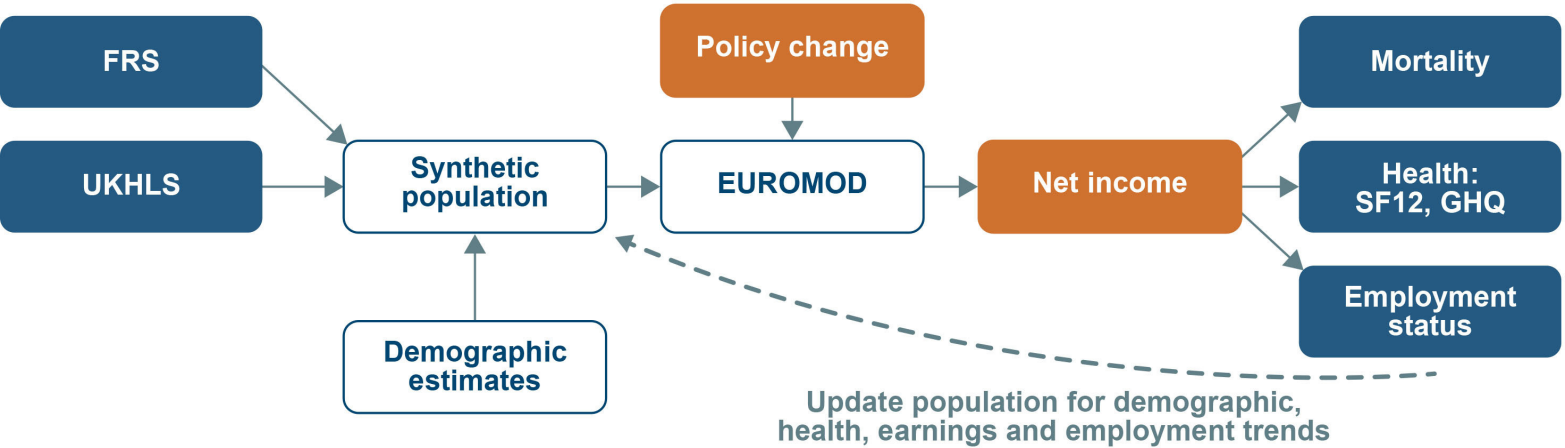
Schematic representation of the Health Equity and Its Economic Determinants microsimulation model for the UK. EUROMOD, a European Union tax-benefit microsimulation model; FRS, Family Resources Survey; GHQ, General Health Questionnaire; SF-12, Short Form 12-Item Survey; UKHLS, UK Household Longitudinal Survey (also referred to as Understanding Society).

Figure 1 outlines the key components and structure of the HEED microsimulation model. The initial characteristics of the synthetic cohort will be defined on the basis of representative datasets with information drawn from the UKHLS, Family Resources Survey (FRS) and population estimates to include employment status, income measures, GHQ-12 score, SF-12 score, education, country of residence and socioeconomic position. In addition to using pre-existing inverse probability weights, integrating multiple surveys will allow us to create a synthetic dataset that is as representative as possible, as well as allowing us to use variables that are derived from different datasets to overcome the limitation that a single dataset may not include all relevant variables.

An age–period–cohort model will be created for new births and deaths, based on the UK Office for National Statistics projections (and devolved equivalents), which accounts for differential risks of these events by age, sex, education, household structure, nation and deprivation. Similarly, trends (stratified by the same subgroups) in the health outcomes, income and employment rates will be assessed using the UKHLS data and used to predict future trends. These models will be used to create an annual probability (which varies year to year) for each individual experiencing these events that will be used to project the cohort forward in annual increments.

To estimate the impact of changes in taxation and/or social security policies on health, we will first estimate potential changes in income and welfare receipts due to policy reforms. EUROMOD (and its UK equivalent, UKMOD) is a widely used open-source, microsimulation model to estimate the impact of changes in tax-benefit policies.^27 43^ It does this by deterministically applying taxation and benefits rules to individual-level repeated cross-sectional data that are available from the European Union Survey on Income and Living Conditions (EU-SILC) or from the FRS for UKMOD. Every year, the EUROMOD/UKMOD teams collate detailed information about the income taxation and social security systems of European countries on an ongoing basis, therefore allowing simulations of the economic impacts for a wide range of policies to be implemented. The required inputs for EUROMOD/UKMOD will be included in the synthetic cohort for HEED to allow for ease of integration. This will allow us to simulate how income is likely to change as a consequence of applying different policies.

In addition to the largely deterministic element of the effect of taxation on income, the impacts on the labour market due to changes in work incentives will also be included in HEED. We will do this by adapting a previously created labour supply model which has been implemented using EUROMOD.^44^ More specifically, we will incorporate a discrete choice random utility maximisation model which estimates the relationship between income and work incentives in order to predict how employment status might change for the following year.^45^

Initially, the HEED microsimulation model will create a projection for the future which incorporates existing trends but no specific policy changes by transitioning the synthetic population forward in 1-year increments, with the following year’s observations informed by the current year’s observations and a secular trend. The microsimulation model will first allow for individuals to potentially die by applying the age–period–cohort model previously described. After accounting for deaths for a given year, health status measures will be predicted for every individual over time, with their predicted values being influenced by their previous value, a secular trend and a stochastic term. Income and employment measures will be similarly predicted. To relate income and employment status to health, we will build on the epidemiological concept of the population impact fraction.^46^ We will consider health outcomes to be composed of a baseline risk not modifiable by the exposures of interest and a proportion that can be.

Several tests to assess the validity of the HEED microsimulation model will be undertaken. Comparison will be made between predictions from HEED and observed data and datasets which were not used in the model’s development (eg, UKHLS years not included in model development, Health Survey for England dataset). We will also consider comparing the simulated impacts of policies on health to policy outcomes which have already been evaluated in natural experiment studies (identified in phase I).

### Phase III: conducting policy simulations for the UK

To investigate the potential impacts of different taxation and social security approaches, a number of realistic policy interventions will be developed with policymakers. These intervention scenarios will be compared against a baseline scenario of current and planned policies. It is anticipated that these scenarios will focus more on revenue-neutral changes, as well as social security reforms with modest revenue changes. UKMOD will be used to help identify revenue-neutral proposals.^47^ This will mean that ‘second-round’ effects (eg, the health impacts of changes in government expenditure) can be more readily ignored. We plan to address this limitation in future work beyond this project.

Examples of policy intervention scenarios that could be investigated in the HEED microsimulation model include

1. Revenue-neutral changes to the income tax system to modify progressiveness.
2. Altering the value of unemployment benefits.
3. Altering the value of incapacity-related benefits.

To model health impacts, we will first assess health outcomes for the baseline scenario (ie, no interventions applied). The income received by the same synthetic population in year 0 will then be modified, based on applying the policy scenario in UKMOD. Future health and economic outcomes will then be projected forward. The predicted impact of the policy intervention will be evaluated by comparing the health (and economic) outcomes for the same population under the intervention scenario against the baseline scenario. Importantly, the use of microsimulation enables disaggregation of the estimated impacts to specific population subgroups of interest, including by age group, sex, education, household structure (eg, single parents vs coupled parents), deprivation and country/region. For socioeconomic measures, we will consider reporting absolute and relative equity slope indices to establish the health equity impacts of any reforms, as well as establishing the extent to which any specific reform could contribute to narrowing overall health inequities.^48^

Key assumptions in the modelling process include the following:

1. Data are representative of the populations of interest, after incorporation of covariates in the sample weighting (ie, missing at random).
2. Effect estimates are unbiased and generalisable, although imprecision from sampling error is accounted for.
3. Behavioural responses to policy changes are assumed to occur within 1 year of changes in policy.
4. Future health gains have the same value as current health gains.
5. Second-round economic impacts are not modelled, including no consideration of the impacts of any changes to taxation revenue.
6. There is assumed to be no additional impact of income inequality or other country-level factors beyond the effects that arise from individuals.
7. No account is taken of potential international migration flows or educational mobility.
8. Projections of future trends are adequately modelled, and in particular, we cannot account for major macroeconomic shocks.

The robustness of our findings to several of these assumptions will be systematically tested. For assumption 1, we will compare the characteristics of the synthetic population to routinely available estimates derived from data sources not used in the creation of our population. Assumptions 1 and 2 reflect several uncertainties in the input parameters for HEED (eg, relative risks for effects on health outcomes, disease burden projections and the extent that effects differ across population subgroups). Probabilistic sensitivity analysis, using second order Monte Carlo simulation, will be used to incorporate this uncertainty, generating 95% uncertainty intervals.^49^ We will investigate whether our results are robust to alternative specifications of effect sizes on the basis of bias analysis.^50^ We will check the robustness of our conclusions by altering inputs that are determined as critical by our policy stakeholders (eg, differing timescales for implementation of policy reforms). There are also structural assumptions that underpin the model which are more difficult to incorporate within probabilistic sensitivity analysis (since the underlying distributions may be unclear). We will therefore compare our results with differing assumptions about the extent of lag periods between income, employment status and the health outcomes of interest. The WHO recommends no discounting of future health gains, but other bodies (such as the UK’s National Institute for Health and Social Care Excellence) recommend applying a discount rate.^51^ We will implement alternative discount rates of 1.5% and 3.5%. The impact of assumption five is minimised through the focus on revenue-neutral policies. Ignoring the additional influence of country-level factors (such as income distribution) is likely to underestimate the effects of any progressive reforms. While the existence of any additional impacts of income inequality remains contentious,^52 53^ we will explore the potential impact any added effects of income inequality on self-rated health and mortality might have.^54^ Since the research was first designed, the emergence of the COVID-19 pandemic has led to major economic shocks. We will attempt to align our estimates to shocks observed in available data, as well as explore different plausible recovery scenarios. In future research, we hope to add further sophistication to HEED to allow assumptions 6–8 to be relaxed. This iterative approach will help ensure feasibility of the work.

### Phase IV: expanding the microsimulation model to include other European countries

As discussed with respect to phase I, the UK datasets (supplemented by registry data from Sweden for mortality and psychotropic prescriptions) allow the study of inter-relationships between health and its economic determinants longitudinally in population-based samples. Unfortunately, comparable data are not currently available in a harmonised form across European Union countries. This lack of comparable longitudinal data will necessitate adaptation of the HEED model to extend to countries other than the UK.

**Figure 2:**
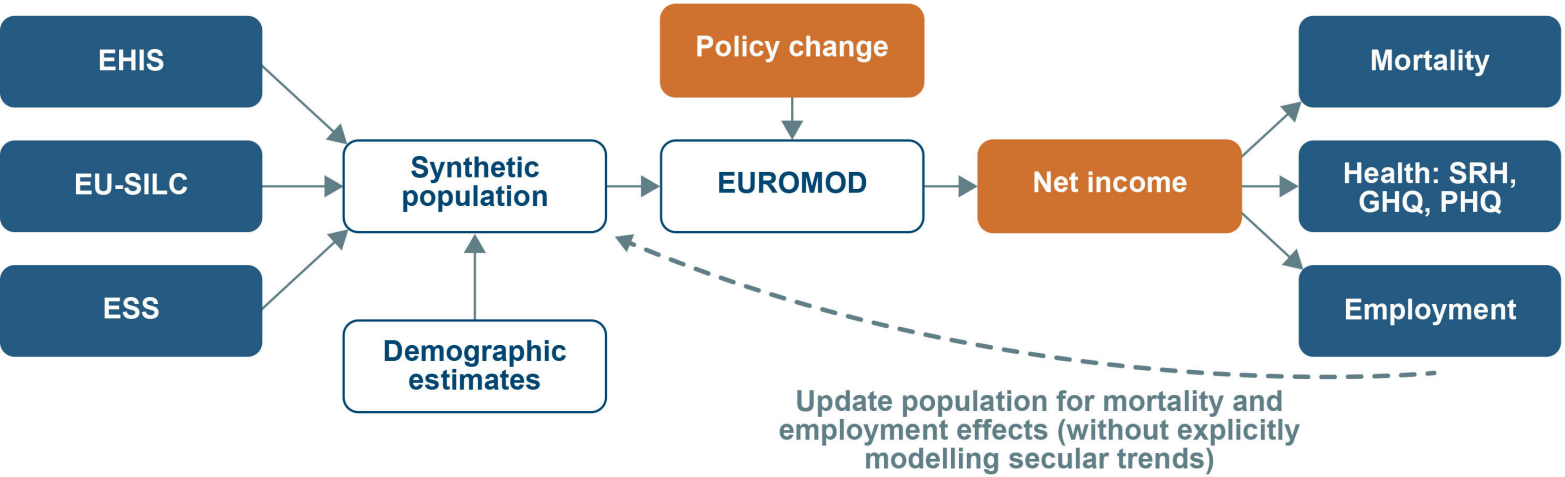
Schematic representation of the Health Equity of Economic Determinants model for European Union countries. EHIS, European Health Interview Survey; ESS, European Social Survey; EU-SILC, European Union Survey on Income and Living Conditions; GHQ, General Health Questionnaire; PHQ, Patient Health Questionnaire; SRH, self-rated health.

Figure 2 outlines the key components and structure of the pan-European HEED microsimulation model. Four European datasets will be drawn on to create synthetic populations for each country of interest, thereby providing a ‘synthetic laboratory’ resource for a wide range of future policy experimentation. Demographic characteristics of the population, as well as sex-specific and age-specific death rates, will be retrieved from the UN World Population Prospects. Health variables will be derived from the European Health Interview Survey (EHIS) and the European Social Survey (ESS). There have been three waves of the EHIS carried out across European Union member states (with data collection in 2006–2009, 2013–2015 and 2019 onwards), although only 17 of the 28 EU countries participated in the first wave. Self-rated health (5-point Likert scale) and the Patient Health Questionnaire 9 (PHQ-9) have been collected in all three waves. Self-rated health is a well-established predictor of all-cause mortality^55 56^ and will be used as the main measure of health status. PHQ-9 is an epidemiological tool to assess the presence of likely depression and will be mapped onto GHQ-12 to allow parameter estimates to be informed by estimates obtained using g-methods. The ESS has been conducted every 2 years from 2002, although not all countries have participated in each wave (eg, Greece, Luxembourg, Malta and Romania did not participate in 2018). Due to the lack of baseline data, these four countries will be excluded. The ESS includes measures of self-rated health, life satisfaction (10-point Likert scale) and GHQ-12 (in 2012 and 2014 only). Estimates of mortality by highest educational attainment have already been produced by cross-national analyses of register data.^57^ The relative risks for mortality across different educational groups will therefore be obtained from these.

To source socioeconomic input parameters, the aforementioned data sources will be supplemented with the EU-SILC. This is a representative survey carried out by member states for the purposes of calculating key economic indicators (eg, unemployment rates) and harmonised by EuroStat after its conduct. It includes information about individual and household employment status, education level, income and household structure. It is also the primary data source used for the existing EUROMOD model.

The pan-European HEED model will relate changes in income to health outcomes in a manner similar to the UK version. However, the lack of longitudinal data across Europe (except for specific age groups, eg, older people in the Survey of Health, Ageing and Retirement in Europe (SHARE)) makes it more difficult to create longer-term projections of health outcomes for the future. Therefore, secular economic and health trends will not be incorporated within the European model. The European HEED model will be limited to time horizons of 1 and 5 years, with a view to studying longer-term impacts in future work.

Several aspects of both income taxation and social security policy across Europe will be simulated and studied using the pan-European HEED microsimulation policy. Relevant intervention scenarios will be coproduced with policy stakeholders in the European Commission, member states and WHO Europe.

Due to the fewer datasets available across multiple European countries, the potential for validation will be more limited than for the UK model. While policy modelling will use the most recently available data, the model will also be fitted using older datasets to estimate predicted values for the most recent period. Similarly, mortality rates for different population subgroups will be predicted by the model and compared with estimates from other data sources (eg, published mortality estimates by education level from national statistical agencies). Lastly, predictions based on the most recent survey waves will be compared with those from SHARE for the subgroup of the population included in both analyses (ie, aged 50–64 years).

### Patient and public involvement

No specific patient involvement is planned. We will work with relevant stakeholders (including third-sector organisations) and disseminate our findings to the public.

## Ethics and dissemination

No further ethical approval will be required for our main analytical datasets, although data access applications may be required.

HEED will make an important contribution to research on the economic determinants of health. The dissemination plan aims to engage academic, policy and public audiences.

Academic publications and conference presentations will cover three main areas:

1. Effect estimates for the economic determinants on health.
2. Model development and methodology for the UK and pan-European microsimulation model.
3. Policy simulations.

We will work with policy stakeholders throughout the project to ensure the utility of HEED to potential policy, as well as academic, end users. Short, accessible policy briefings will be targeted at relevant civil servants (eg, European Commission and Public Health Scotland). Broader public engagement and debate will be achieved through mass media and a project website.

The HEED model will use widely available software, such as R or JAVA. Both the syntax and underlying synthetic data will be made freely available through GitHub and the project website, facilitating transparency and enabling reuse by other researchers and policymakers.

## Supplementary Material

Reviewer comments
