# Supplementary material for: Health Equity and Its Economic Determinants (HEED): protocol for a pan-European microsimulation model for health impacts of income and social security policies
Source: BMJ Open. 2022 Jul 19;12(7):e062405. doi: 10.1136/bmjopen-2022-062405 (PMC9301818; doi:10.1136/bmjopen-2022-062405)
Supplement: Reviewer comments [file bmjopen-2022-062405.reviewer_comments.pdf]

PEER REVIEW HISTORY

BMJ Open publishes all reviews undertaken for accepted manuscripts. Reviewers are asked to complete a checklist review form (**http://bmjopen.bmj.com/site/about/resources/checklist.pdf**) and are provided with free text boxes to elaborate on their assessment. These free text comments are reproduced below.

ARTICLE DETAILS

**TITLE (PROVISIONAL)**	Protocol for a Pan-European Microsimulation Model for Health impacts of Income and Social Security Policies: Health Equity and its Economic Determinants (HEED)
**AUTHORS**	Katikireddi, Srinivasa; Kopasker, Daniel; Pearce, Anna; Leyland, Alastair; Rostila, Mikael; Richiardi, Matteo

This article was not externally reviewed at BMJ Open. Protocols that have been independently assessed prior to submission to BMJ Open are usually fast-tracked to publication on the grounds that further substantial changes will not be possible. This independent assessment will usually be external, independent review for both a substantial grant award from a non-commercial or government funder and ethics approval.
